# Nested Grover’s Algorithm for Tree Search

**DOI:** 10.3390/e28010024

**Published:** 2025-12-24

**Authors:** Andreas Wichert

**Affiliations:** Department of Computer Science and Engineering, INESC-ID & Instituto Superior Técnico, University of Lisbon, 2740-122 Porto Salvo, Portugal; andreas.wichert@tecnico.ulisboa.pt

**Keywords:** quantum tree search, Grover’s algorithm, heuristics, nested search

## Abstract

We investigate optimizing quantum tree search algorithms by employing a nested Grover Algorithm. This approach seeks to enhance results compared to previous Grover-based methods by expanding the tree of partial assignments to a specific depth and conducting a quantum search within the subset of remaining assignments. The study explores the implications and constraints of this approach, providing a foundation for quantum artificial intelligence applications. Instead of utilizing conventional heuristic functions that are incompatible with quantum tree search, we introduce the partial candidate solution, which indicates a node at a specific depth of the tree. By employing such a function, we define the concatenated oracle, which enables us to decompose the quantum tree search using Grover’s algorithm. With a branching factor of 2 and a depth of *m*, the costs of Grover’s algorithm are $O(2^{m/2})$. The concatenated oracle allows us to reduce the cost to $O(m\cdot 2^{m/4})$ for *m* partial candidate solutions.

## 1. Introduction

We investigate the optimization of quantum tree search algorithms by employing a nested Grover Algorithm. We explore the implications of this approach and identify the resulting constraints. Our findings demonstrate the potential for enhanced results compared to previously developed Grover-based methods. Instead of adopting the hybrid divide-and-conquer methods that are predicated on quantum backtracking and quantum walk, as indicated in [1,2], we will proceed with the initial conception of a nested version of Grover’s nested search, as described in [3]. In a nested version of Grover search, the complete tree of partial assignments is expanded to a specific depth, and then a quantum search is conducted within the subset of partial assignments that have not yet been eliminated.

It is claimed that, for certain reasonable distributions of problems, the average complexity of the original nested version of Grover search [3] will be less than that obtained from Grover search. This claim is supported by references [1,3]. Building upon the initial concept of a nested version of Grover search, we will adopt a combinatorial approach to address the limitations inherent in the original work, as described in [3]. For simplicity, we assume that the tree is uniform and its size is known beforehand. We will demonstrate our approach on simple examples using Qiskit, see [4]. The main contributions of this paper are as follows:

- We introduce the definition of a partial candidate solution and the contented oracles.
- We analyze the constraints of the original Grover’s nested search as introduced in [3];
- We introduce the iterative approach with a quadratic speedup in comparison to the original Grover’s algorithm;
- We investigate the possibilities of dividing the original space into two disentanglement subspaces and the resulting consequences;
- We introduce the concept of performing a permutation on a subspace and the corresponding constraint.

Our approach imposes specific constraints that elucidate the concepts of partial candidate solutions within the context of quantum tree search. This work provides the foundation for quantum artificial intelligence applications, including quantum problem-solving and quantum production systems.

## 2. Quantum Tree Search

**Figure 1:**
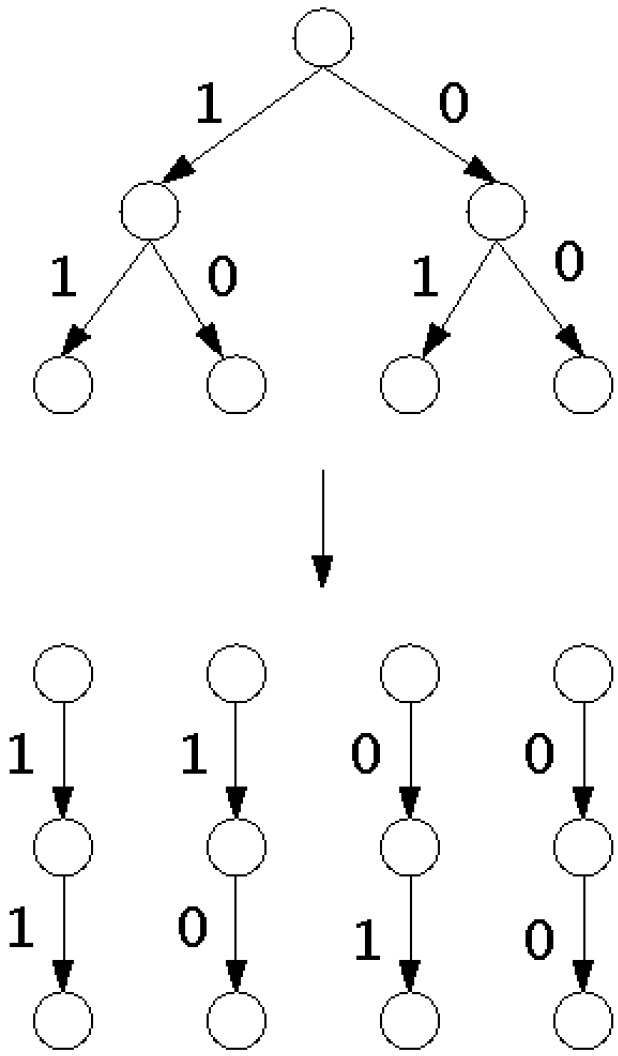
Search tree for b=2 and depth m=2. Each question can be represented by a bit. Each binary number (11, 10, 01, 00) represents a path descriptor *m* from the root to the leaf.

In this section, we outline the application of Grover’s algorithm to the tree search problem. Nodes and edges in a search tree represent states and transitions between states [5]. The initial state is the root, and from each state, either *B* states can be reached or it is a leaf. From a leaf no other state can be reached. *b* represents the branching factor of a node, indicating the number of possible choices. A leaf can either be the goal of the computation or an impasse when there are no valid transitions to a succeeding state. Each node except the root has a unique parent node, which is called the parent. Every node and its parent are connected by an edge. Each parent has *b* children. If b=2 and the depth of the tree is *m*, each of the *m* questions has a yes/no reply and can be represented by a bit (as shown in Figure 1). The *m* answers are collectively represented by a binary number of length *m*.

**Figure 2:**
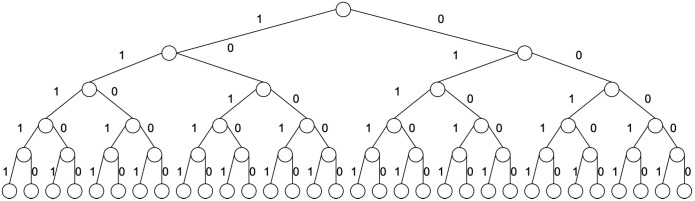
Search tree for b=2 and depth m=5. Each question can be represented by a bit.

There are $n=2^{m}=b^{m}$ possible binary numbers of length *m*. Each binary number represents a path from the root to a leaf. For each goal, a specific binary number indicates the solution. This binary number is called a path descriptor, since it describes a path from the root of the tree to the leaf, see Figure 2 with b=2 and depth m=5.

For a constant branching factor b>2, each question has *b* possible answers. The *m* answers can be represented by a base-*b* number with *m* digits. In a quantum computation, we can simultaneously represent all possible path descriptors. There is one path descriptor for each leaf off the tree. Using Grover’s algorithm, we search through all possible paths and verify whether each path leads to the goal state. This type of procedure is known as a quantum tree search [6]. For $n=b^{m}$ possible paths, the costs are (approximately) $\sqrt{n}=b^{\frac{m}{2}}$, corresponding to the reduced branching factor $b_{q}=\sqrt{b}$.

### Grover’s Algorithm

For a function o(x)

(1) $$o_{\xi }\left(x\right)=\left\{\begin{array}{c} 1\;\;\;if\;\;\;x=\xi \\ 0\;\;\;else \end{array}\right.$$

we seek to identify the value of *x* representing a path descriptor for which o(x)=1, where x=ξ, the path descriptor leading to the solution. This task is equivalent to a decision problem with a binary answer: 1 indicating a successful search and 0 indicating an unsuccessful search. The instance *x* is the input to the problem.

Grover’s amplification algorithm implements exhaustive search in $O(\sqrt{n})$ steps in an *n*-dimensional Hilbert space [7,8,9,10]. This algorithm is derived from the Householder reflection of the state |x〉 of *m* qubits, where $n=2^{m}$. Grover’s amplification algorithm is optimal for exhaustive search, as demonstrated by the lower bound $\Omega (\sqrt{n})$ established by [11]. One can prove that a better algorithm cannot exist [12,13]. It follows that using a quantum computer, *NP-complete* problems remain *NP-complete*. Grover’s amplification algorithm provides a quadratic speedup over classical computers, which would require *n* steps to solve the problem.

For *k* solutions and more than two qubits (m>2, $n=2^{m}$) the number *r* of iterations is the largest integer

(2) $$r=\left\lfloor \frac{\pi }{4}\cdot \sqrt{\frac{2^{m}}{k}}\right\rfloor .$$

The value of *r* depends on the relation of *n* versus *k*. For n=4 and k=1 we need only one rotation, and we also need only one rotation for

$$\frac{n}{4}=k$$

to find **one** of the *k* solutions. The iterative amplification algorithm requires the value of *k* in order to determine the number of iterations. We can determine the value of *k* by the quantum counting algorithm [14,15,16].

Uniform distributions are essential for Grover’s algorithm. If the distribution is non-uniform, the algorithm may not function properly or require adaptation which leads to the same complexity $O(\sqrt{n})$ [3,17,18,19]. Consequently, we cannot simply mark potential solutions by assigning higher amplitude values to speed up the search. For instance, an adapted algorithm for sparse distributions where the majority of amplitudes are zero must generate a uniform distribution for the unmarked sets, with the resulting complexity $O(\sqrt{n})$ [17,18,19].

In our analysis in relation to the *O* notation $O(\sqrt{\frac{n}{k}})$ and for simplification, we will assume the approximate complexity of Grover’s algorithm as

(3) $$\sqrt{\frac{2^{m}}{k}}=\sqrt{\frac{n}{k}}.$$

instead of using the accurate Equation (2).

## 3. Nested Grover’s Search

Is it possible to integrate nested Grover’s search techniques into the quantum tree search algorithm to enhance the search process? Can the nested Grover’s search be based on heuristic functions? A heuristic function μ(y) estimates the cheapest cost from a node to the goal. The local path descriptor *y* describes the path from the root to the node. Therefore, each node in the tree can be described by a local path descriptor. However, we cannot employ a heuristic function to generate a non-uniform distribution, as it does not confer any advantage in Grover’s search. Furthermore, we cannot compare branches represented by distinct path descriptors. This is also the reason why the min-max algorithm cannot be applied to quantum tree search in games. The reason for this is that distinct branches correspond to distinct superpositions, and these superpositions interact solely through interference.

### 3.1. Partial Candidate Solution

Within the context of quantum tree search, instead of employing a heuristic function μ(y), we will utilize the function h(y), which represents a partial candidate solution, and *y* denotes a path from the root to the corresponding node. In contrast to the heuristic function μ(y), which can mark multiple nodes, h(y) should exclusively mark a single partial solution that includes the node indicated by the path *y*.

### 3.2. Decomposition

**Figure 3:**
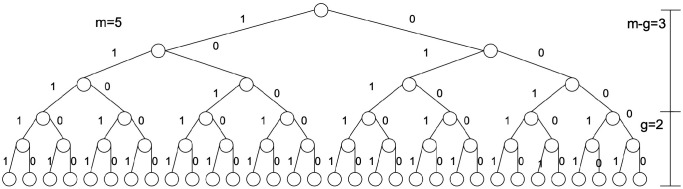
The decomposition of the Hilbert space H of dimension $n=2^{m}$ into subspaces L and U of dimension $2^{g}$ and $2^{m-g}$. In our example, m=5 and g=2.

The idea of saving the costs is based on the inequality that represents the decomposition of the Hilbert space H of dimension $n=2^{m}$ into subspaces L and U of dimension $2^{g}$ and $2^{m-g}$, see Figure 3. For the value *g* we assume

2≤g<m.

The costs of Grover’s algorithm for the quantum tree search algorithm are

$$\sqrt{2^{m}}=\sqrt{2^{g}}\cdot \sqrt{2^{m-g}}.$$

However, the costs associated with Grover’s algorithm for the quantum tree of each subspace would be significantly reduced to

$$\sqrt{2^{g}}+\sqrt{2^{m-g}}$$

(see Figure 4) with

(4) $$\sqrt{2^{g}}+\sqrt{2^{m-g}}<\sqrt{2^{g}}\cdot \sqrt{2^{m-g}},$$

since

$$1<\sqrt{2^{g}}\cdot \sqrt{2^{m-g}}-\sqrt{2^{g}}+\sqrt{2^{m-g}}+1$$

$$1<\left(\sqrt{2^{g}}-1\right)\cdot \left(1-\sqrt{2^{m-g}}\right)$$

because

$$1<\left(\sqrt{2^{g}}-1\right),\;\;1<\left(1-\sqrt{2^{m-g}}\right).$$

### 3.3. Nested Grover’s Search and Entanglement

**Figure 5:**
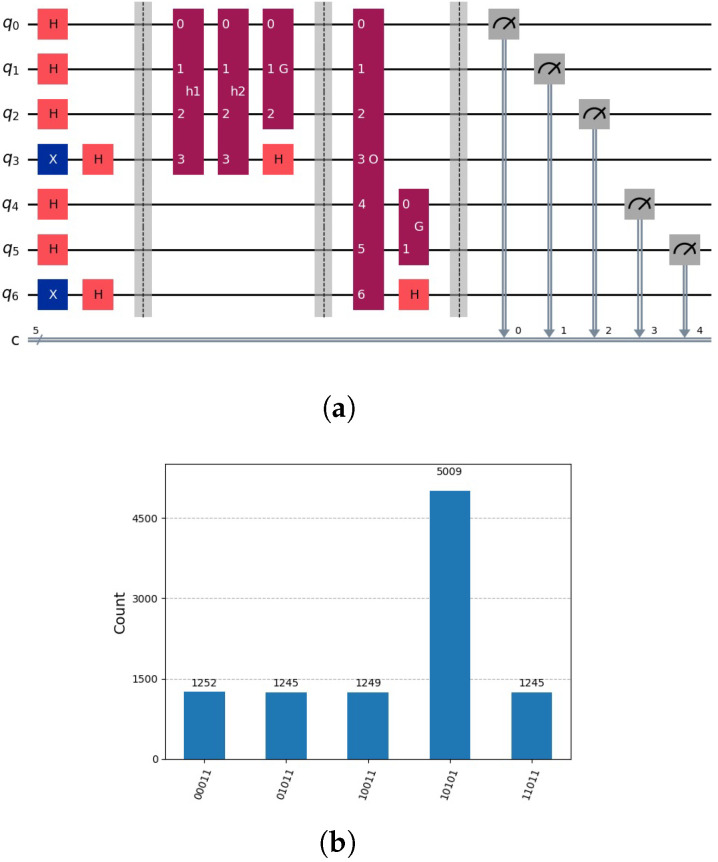
(**a**) Circuit using Grover’s amplification representing oracles $h^{\left(1\right)}\left(y\right)=\neg x_{3}\wedge x_{2}\wedge x_{1}$, $h^{\left(2\right)}\left(y\right)=x_{3}\wedge \neg x_{2}\wedge x_{1}$ for lower qubits and the global oracle $o\left(x\right)=x_{5}\wedge \neg x_{4}\wedge x_{3}\wedge \neg x_{2}\wedge x_{1}$. Note, qubits 3 and 5 are auxiliary qubits used in Grover’s algorithm to mark the solution. We will employ the little-endian notation as used in Qiskit, see [4]. Notation: *H* indicates Hadamard gate, *X* indicates NOT Gate, *h1* and *h2* oracle circuits that marks the solution in the auxiliary qubits. *O* represents the global oracle circuits and *G* Grover’s algorithm circuits. Composed quantum circuits are indicated dark red, quantum gates with colours orange and blue. (**b**) Due to the entanglement of the lower qubits with the upper qubits, the solution indicated by 10101 is indicated only approximately by 50%.

Following, alternatively, the idea that Grover should speed up the search on Hilbert space H of dimension $n=2^{m}$, a set *ℵ* of *v* partial candidate solutions

$$h^{\left(k\right)}\left(y\right)\;\;with\;k\in \left\{1,2,\cdots ,v\right\}$$

is defined that acts on *g* lower qubits with

(5) $$h^{\left(k\right)}\left(y\right)=\left\{\begin{array}{c} 1\;\;\;if\;\;\;y=h_{k}\\ 0\;\;\;else \end{array}\right.$$

on the subspace L of dimension $2^{g}$, with *y* being a local path descriptor. Throughout the paper, we will employ the little-endian notation, which stores the least-significant byte at the smallest address. Qubits are represented from the most significant bit (MSB) on the left to the least significant bit (LSB) on the right (little-endian), similar to binary numbers or bit-string representation on classical computers. We illustrate the fundamental concept of nested Grover search through a simple SAT example, characterized by v=2, g=3, and m=5.

$$h^{\left(1\right)}\left(y\right)=\neg x_{3}\wedge x_{2}\wedge x_{1},\;\;h^{\left(2\right)}\left(y\right)=x_{3}\wedge \neg x_{2},\wedge x_{1}$$

$$y=x_{3},x_{2},x_{1};\;\;x_{k}\in 0,1,$$

$$o\left(x\right)=x_{5}\wedge \neg x_{4}\wedge x_{3}\wedge \neg x_{2}\wedge x_{1},\;\;\;x=x_{5},x_{4},\cdots ,x_{1};$$

$$x_{k}\in \left\{0,1\right\},$$

as indicated by the circuit in Figure 5a, with the results of measurement shown in Figure 5b. In the initial step, we determine the set *ℵ* using Grover’s algorithm on the subspace L. In the subsequent step, we anticipate utilizing this set to expedite the search for the solution indicated by the oracle $o_{\xi }\left(x\right)$, which operates on *m* qubits. Grover’s amplification is applied to the subspace U defined by the upper m−g qubits. However, due to entanglement, we are unable to employ Grover’s algorithm on the upper subspace U defined by m−g upper qubits, as both subspaces L and U become entangled.

To comprehend the behavior we introduce concatenated oracles.

### 3.4. Concatenated Oracles

Concatenation is an associative operation but not a commutative operation. We use for the operation concatenation the specific symbol ‖. If we assume that *x* is represented by *m* bits with the notation

$$x=x_{m},x_{m-1},x_{m-2}\cdots ,x_{2},x_{1};\;\;\;x_{k}\in \left\{0,1\right\},$$

then concatenation is often simply the placement of the binary numbers next to each other,

$$\left(x_{m},x_{m-1},\cdots ,x_{g}\right)\|\left(x_{g-1},\cdots ,x_{1}\right)=x_{m},x_{m-1},\cdots ,x_{2},x_{1}$$

For example, for a binary number of 10 and 11 we get

10 ‖ 11=1011.

We extend the concatenation for the oracle function

$$o_{\xi }\left(x_{m},x_{m-1},x_{m-2}\cdots ,x_{1}\right)=$$

(6) $$u\left(x_{m},x_{m-1},\cdots ,x_{g}\right)\|\;l=\left(x_{g-1},\cdots ,x_{1}\right).$$

Using compact notation,

$$z=\left(x_{m},x_{m-1},\cdots ,x_{g}\right),\;\;y=\left(x_{g-1},\cdots ,x_{1}\right),$$

$$x=\left(x_{m},x_{m-1},\cdots ,x_{g}\right)\|\left(x_{g-1},\cdots ,x_{1}\right),$$

x=z ‖ y,

and the oracle function

$$o_{\xi }\left(x\right)=u\left(z\right)\|\;l\left(y\right)$$

with

$$o_{\xi }\left(x\right)=\left\{\begin{array}{c} 1\;\;\;if\;\;\;x=\xi \\ 0\;\;\;else \end{array}\right.$$

(7) $$u\left(z\right)=\left\{\begin{array}{c} 1\;\;\;if\;\;\;z=u\\ 0\;\;\;else \end{array}\right.\;l\left(y\right)=\left\{\begin{array}{c} 1\;\;\;if\;\;\;y=l\\ 0\;\;\;else \end{array}\right.$$

solution being

ξ=u ‖ l,

with u=u(z) being the upper part of the oracle function that acts on the upper qubits, describing the upper subspace U of the dimension $2^{\left(m-g\right)}$ and l=l(y) being the lower part of the oracle function that acts on the lower qubits, describing the lower subspace L of the dimension $2^{g}$ with

o(x)=o(z,y)=u(z) ‖ l(y).

**Figure 6:**
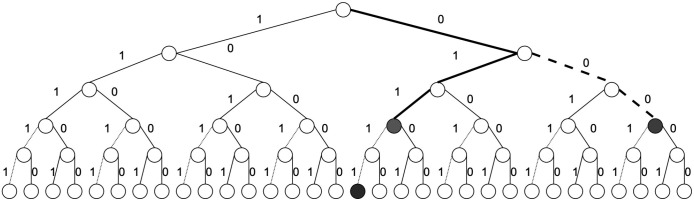
Search tree for B=2 and depth m=5. With $h_{1}=011$, $h_{2}=000$, u=11, l=011 and ξ=01111. Bold lines denote the path to the solution. The dotted line represents the path to the partial candidate solution that does not lead to a solution. Upper-field circles signify partial candidate solutions, while the bold circle in the leaf signifies the solution, which is composed of $h_{1}=l$ and *u*.

Note that l(y) and u(z) do not indicate partial candidate solutions; they are exact copies of the oracle function, and it can be challenging to specify them precisely. Also, *u* indicates the path descriptor. An example is represented in Figure 6.

In the case we constraint *y* values to some values $h_{k}$ with

$$y\in \{h_{1},h_{2},\cdots ,h_{v}\}$$

the constraint oracle can be expressed as $l=h_{k}$ with

$$o\left(x\right)=o\left(z,h_{k}\right)=u\left(z\right)\;\|\;l\left(h_{k}\right).$$

### 3.5. Not Entangled Subspaces

Imagine we know $u_{\mu }\left(z\right)$ that acts on the upper subspace U defined by (m−g) lower qubits and given set *ℵ* of *t* partial candidate solutions that act on the subspace L defined by upper *g* qubits,

$$h^{\left(k\right)}\left(y\right)\;\;with\;k\in \left\{1,2,\cdots ,v\right\}.$$

If we apply both oracles and use Grover’s algorithm to each Hilbert space defined by the subspace L and U, the results are not entangled since both subspaces are not entangled. In the first step, we determine *ℵ* using Grover’s algorithm on the Hilbert space defined by the subspace L. In the second step, we determine u(z) using Grover’s algorithm on the upper qubits of the upper subspace U with

(8) H=U⊗L.

If we measure both subspaces, we see that each solution in the lower subspace L defines a new combination of the one solution in the upper subspace U. One of the combination could correspond to the global solution of the space as described by the oracle $o_{\xi }\left(x\right)$, however we do not know if this is the case.

**Figure 7:**
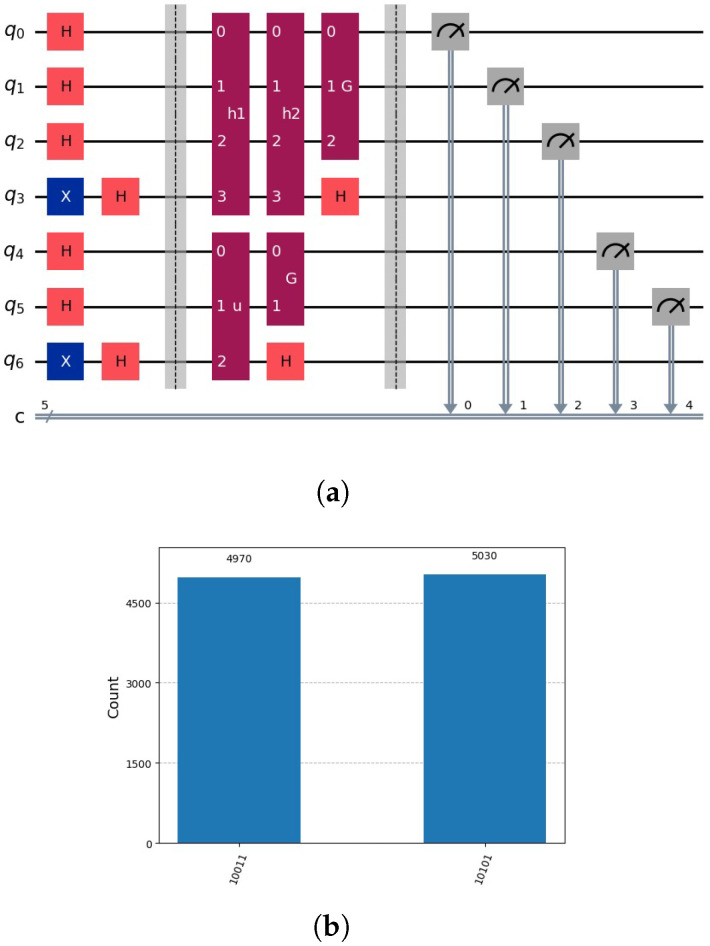
(**a**) Circuit employing Grover’s amplification to represent oracles $h^{\left(1\right)}\left(z\right)=\neg x_{3}\wedge x_{2}\wedge x_{1}$, $h^{\left(2\right)}\left(y\right)=x_{3}\wedge \neg x_{2}\wedge x_{1}$ for lower qubits, and the oracle for the upper qubits $u\left(z\right)=x_{5}\wedge \neg x_{4}$. When we measure, we are not guaranteed to obtain the solution ξ=o(x), since o(x) was not checked and the possible combinations may be not present. Notation: *H* indicates Hadamard gate, *X* indicates NOT Gate, *h1* and *h2* partial candidate solution circuits that marks the solution in the auxiliary qubit, *u* represents upper part of the oracle function circuit and *G* Grover’s algorithm circuits. Composed quantum circuits are indicated dark red, quantum gates with colours orange and blue. (**b**) Due to the combinatorial problem that results in our case, we yield two solutions with equal probability, corresponding to the number of partial candidate solutions, since (v=2) with $\frac{1}{\sqrt{v}}\sum_{k=1}^{v}|u,h_{k}\rangle =\frac{1}{\sqrt{2}}\cdot (|10011\rangle +\left|10101\rangle \right)$.

For example in the preceding example we introduce with v=2 and g=3

$$h^{\left(1\right)}\left(y\right)=\neg x_{3}\wedge x_{2}\wedge x_{1},\;\;h^{\left(2\right)}\left(z\right)=x_{3}\wedge \neg x_{2}\wedge x_{1}$$

and the oracle for the (5−3) upper qubits

$$u\left(z\right)=x_{5}\wedge \neg x_{4}$$

as indicated by the circuit in Figure 7a, with the results of measurement shown in Figure 7b.

The approach can be broadly characterized as

(9) $$\frac{1}{\sqrt{m-g}}\cdot \sum_{z\in B^{m}}{\left(-1\right)}^{u(z)}\cdot |z\rangle ,\;\;\frac{1}{\sqrt{g}}\cdot \sum_{y\in B^{g}}{\left(-1\right)}^{h^{\left(k\right)}\left(y\right)}\cdot |y\rangle$$

(10) $$\left(\frac{1}{\sqrt{m-g}}\cdot \sum_{z\in B^{m}}{\left(-1\right)}^{u(z)}\cdot |z\rangle \right)⊗\left(\frac{1}{\sqrt{g}}\cdot \sum_{y\in B^{g}}{\left(-1\right)}^{h^{\left(k\right)}\left(y\right)}\cdot |y\rangle \right)$$

after applying Grover’s algorithm to subspace L

(11) $$\left(\frac{1}{\sqrt{m-g}}\cdot \sum_{z\in B^{m}}{\left(-1\right)}^{u(z)}\cdot |z\rangle \right)⊗\left(\frac{1}{\sqrt{v}}\sum_{k=1}^{v}|h_{k}\rangle \right)$$

and the subspace U

(12) $$|u\rangle ⊗\left(\frac{1}{\sqrt{v}}\sum_{i=k}^{v}|h_{k}\rangle \right)=\frac{1}{\sqrt{v}}\sum_{k=1}^{v}|u,h_{k}\rangle$$

since both subspaces L and U are not entangled.

### 3.6. Entangled Subspaces

In original nested Grover’s search (see Section 3.3), after applying Grover’s algorithm to the subspace L we get

(13) $$\left(\frac{1}{\sqrt{m-g}}\cdot \sum_{z\in B^{\left(m-g\right)}}|z\rangle \right)⊗\left(\frac{1}{\sqrt{v}}\sum_{k=1}^{v}|h_{k}\rangle \right)$$

both subspaces L and U are entangled because

|x〉=|z,y〉,

$$o\left(x\right)=o\left(z,y\right)=o\left(z,h_{k}\right)=u\left(z\right)\;\|\;h^{\left(k\right)}\left(y\right)=u\left(z\right)\;\|\;l\left(y\right),$$

(14) $$\left(\frac{1}{\sqrt{m-g}}\cdot \frac{1}{\sqrt{v}}\cdot \sum_{z\in B^{\left(m-g\right)}}\sum_{k=1}^{v}{\left(-1\right)}^{o(z,h_{k})}\cdot |z,h_{k}\rangle \right).$$

Applying Grover’s algorithm to the subspace U results in the creation of entanglement.

(15) $$\left(\frac{1}{\sqrt{m-g}}\cdot \frac{1}{\sqrt{v}}\cdot \sum_{z\neq u}\sum_{h_{k}\neq l}|z,h_{k}\rangle \right)+\frac{1}{\sqrt{v}}|\xi \rangle .$$

In our preceding example with v=2 (see Figure 5) after applying Grover’s algorithm to the subspace L

(16) $$\frac{1}{2}\cdot \frac{1}{\sqrt{2}}\cdot \sum_{z\in B^{2}}\sum_{k=1}^{2}{\left(-1\right)}^{o(z,h_{2})}\cdot |z,h_{k}\rangle$$

(17) $$\left(\frac{1}{\sqrt{8}}\cdot \sum_{z\in B^{2}}{\left(-1\right)}^{o(z,h_{2})}\cdot |z,h_{1}\rangle \right)+\left(\frac{1}{\sqrt{8}}\cdot \sum_{z\in B^{2}}{\left(-1\right)}^{o(z,h_{2})}\cdot |z,h_{2}\rangle \right)$$

(18) $$\left(\frac{1}{\sqrt{8}}\cdot \sum_{z\in B^{2}}|z,h_{1}\rangle \right)+\left(\frac{1}{\sqrt{8}}\cdot \sum_{z\in B^{2}}{\left(-1\right)}^{o(z,h_{2})}\cdot |z,h_{2}\rangle \right)$$

After applying Grover’s algorithm to the subspace U we get

(19) $$\left(\frac{1}{\sqrt{8}}\cdot \sum_{z\in B^{2}}|z,h_{1}\rangle \right)+\frac{1}{\sqrt{2}}\cdot |\xi \rangle .$$

with the state

$$\;\frac{1}{\sqrt{8}}\cdot \left(|00,h_{1}\rangle +|01,h_{1}\rangle +|10,h_{1}\rangle +|11,h_{1}\rangle \right)+$$

$$\frac{1}{\sqrt{8}}\cdot \left(|00,h_{2}\rangle -|01,h_{2}\rangle +|10,h_{2}\rangle +|11,h_{2}\rangle \right),$$

After applying Grover’s algorithm to the subspace U we get

$$\frac{1}{\sqrt{8}}\cdot \left(|00,h_{1}\rangle +|01,h_{1}\rangle +|10,h_{1}\rangle +|11,h_{1}\rangle \right)+\frac{1}{\sqrt{2}}\cdot |\xi \rangle ,$$

see Figure 5b. Grover’s algorithm operates on the initial two qubits (upper qubits). It operates on two subspaces of U, one for $h_{1}$ and the other for $h_{2}$. In the subspace defined by $h_{1}$, there is no marking. In the other subspace, there is a marking. As a result of Grover’s amplification, the first subspace exhibits a uniform distribution, while the second subspace contains a marking indicating the solution. Each of the two subspaces defined by $h_{1}$ and defined by $h_{2}$ has a probability of being measured of 0.5. In the first subspace, the uniform distribution of each state is represented by 0.5/4=1/8=0.125. In the second subspace, the solution is represented by 0.5, as indicated in Figure 5b. The number of partial candidate solutions, denoted as *v*, is inversely proportional to the probability of measuring the solution, which is represented as 1/v. How can this problem be effectively addressed? Two potential solutions to this problem are the iterative approach and the idea of the disentanglement of the two subspaces L and U.

## 4. The Iterative Approach

**Figure 8:**
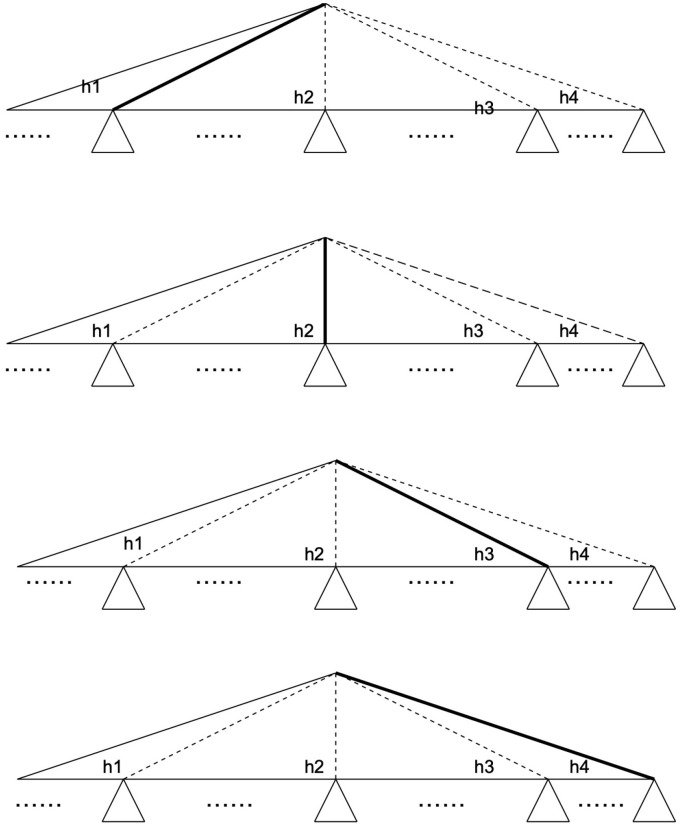
Example for v=4. Determine the solution $h_{1}$ by Grover’s algorithm on the subspace L. Verify if the solution indicated by the oracle $o_{\xi }\left(x\right)$ exists. Repeat the procedure for $h_{2},h_{3},h_{4}.$ The bold line signifies the verification of the partial candidate solution.

With the set *ℵ* of *v* partial candidate solutions

$$h^{\left(k\right)}\left(y\right)=h_{k}\;\;with\;k\in \left\{1,2,\cdots ,v\right\}$$

and the global oracle $o_{\xi }\left(x\right)$, we built a circuit with one possible could-be solution of the set *ℵ* and the global oracle. We determine in the first step one solution of one $h^{\left(k\right)}\left(y\right)$ using Grover’s algorithm on the subspace L, and in the second step we the global oracle $o_{\xi }\left(x\right)$ acts on *m* qubits. Subsequently, we apply Grover’s amplification to the subspace U, which is defined by the upper m−g qubits. Figure 8 indicates an example representing the decomposed search tree for v=4.

**Figure 9:**
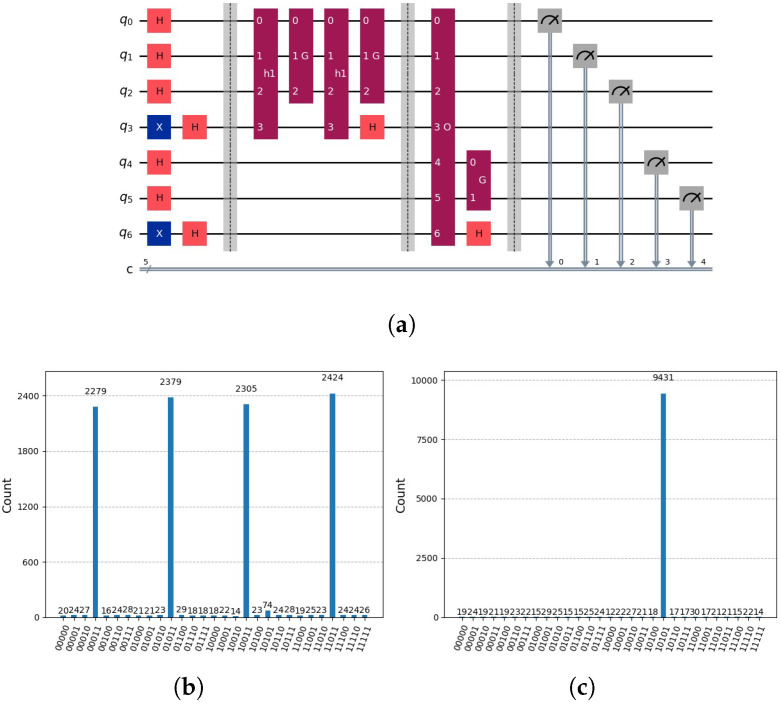
(**a**) Circuit using Grover’s amplification representing oracle $h^{\left(1\right)}\left(y\right)=\neg x_{3}\wedge x_{2}\wedge x_{1}$ for lower qubits and the global oracle $o\left(x\right)=x_{5}\wedge \neg x_{4}\wedge x_{3}\wedge \neg x_{2}\wedge x_{1}$. Notation: *H* indicates Hadamard gate, *X* indicates NOT Gate, *h1* and *h2* partial candidate solution circuits that marks the solution in the auxiliary qubits. *O* represents the global oracle circuits and *G* Grover’s algorithm circuits. Composed quantum circuits are indicated dark red, quantum gates with colours orange and blue. (**b**) Since the solution does not exist, when measuring we get the combination of $h_{1}$ with all four possible combinations of the two upper qubits $x_{5}$ and $x_{6}$ with the probability 1/8. (**c**) The solution for a circuit ξ=u ‖ l with $l=h_{2}$. It is the same circuit as shown in (**a**), only $h^{\left(1\right)}\left(y\right)$ is replaced with $h^{\left(2\right)}\left(z\right)=x_{3}\wedge \neg x_{2}\wedge x_{1}$.

If a solution exists, the subspaces L and U are not entangled with H=U⊗L. In this case, by measuring H, we obtain the solution. This is because the number of partial candidate solutions represented in our circuit is “one,” $v^{*}=1,$ and the probability of measuring a solution, if it exists, is $1/v^{*}=1.$ We measure the state and test whether a solution is present. If it is, we terminate the process; otherwise, we select another potential solution from the set ℵ. For the simple preceding SAT example with v=2 and g=3 and m=5 we get two circuits, see Figure 9.

### 4.1. Algorithm

Given set *ℵ* of *v* partial candidate solutions

$$h^{\left(k\right)}\left(y\right)=h_{k}\;\;with\;k\in \left\{1,2,\cdots ,v\right\}$$

and the global oracle $o_{\xi }\left(x\right)$.

For k=1 to *v*:

1. For the could-be solution $h^{\left(k\right)}\left(y\right)=h_{k}$ build a circuit;
2. Determine the solution $h_{k}$ by Grover algorithm on the subspace L;
3. Verify if the solution indicated by the oracle $o_{\xi }\left(x\right)$ exists;
4. If solution exists success, exit the loop, otherwise, continue the loop.

Since both subspaces L and U are not entangled, if a solution exists, it is readily apparent. We measure the state and verify whether it is a valid solution. If it is a solution, we conclude the process. Otherwise, we select another partial candidate solution from the set *ℵ*.

### 4.2. Optimal Dimension

How can we choose an optimal dimension of the two subspaces U and L? The optimal dimension of the two subspaces results in the minimal costs represented by the minimum of the function

(20) $$\sqrt{2^{m\cdot (1-a)}}+\sqrt{2^{m\cdot a}}\;\;with\;a\in \left[0,1\right]$$

which is equivalent to

$$e^{m\cdot (1-a)}+e^{m\cdot a}\;\;with\;a\in \left[0,1\right]$$

(21) $$\frac{\partial }{\partial a}\cdot \left(e^{m\cdot (1-a)}+e^{m\cdot a}\right)=-e^{m\cdot (1-a)}\cdot m+e^{m\cdot a}\cdot m;\;\;a\in \left[0,1\right]$$

with the solution

$$a=\frac{1}{2}.$$

### 4.3. Costs

Within the subspace U, the costs associated with Grover’s algorithm are $\sqrt{2^{m/2}}$, while within the subspace L, the corresponding costs remain $\sqrt{2^{m/2}}$. We need to verify the proposed solution leads to a global solution. Subsequently, we must repeat the procedure in the worst case *v* times, leading to the cost

(22) $$v\cdot \left(\sqrt{2^{m/2}}+\sqrt{2^{m/2}}+1\right)=v\cdot \left(2\cdot 2^{m/4}+1\right)$$

with the constraint for *v* that the cost are below $2^{m/2}$ with

(23) $$v<\frac{n^{1/2}}{2\cdot n^{1/4}+1}\approx n^{1/4}=2^{m/4}.$$

since $n=2^{m}$. We observe that maximal possible value for *v* is approximately equal to the number of nodes located at one-fourth of the depth of the search tree. We can express the cost of iterative search it in *O* notation, given v≈m, as

(24) $$O\left(v\cdot n^{1/4}\right)=O\left(\log n\cdot n^{1/4}\right).$$

## 5. Disentanglement of L and U

Can we disentangle the spaces L and U with H=U⊗L by knowing the function u(z)? We test the global oracle $o_{\xi }\left(x\right)$ over the whole space of dimension *m* that defines the Hilbert space $n=2^{m}$. The test should not entangle both subspaces L and U. We rely on the assumption that ξ exists and can be decomposed as

ξ=u ‖ l.

Knowing $o_{\xi }\left(x\right)$, assuming the u(z) is correct and with set *ℵ* the task is to verify if

$$o\left(x\right)=o\left(z,h_{k}\right)=u\left(z\right)\|\;l\left(h_{k}\right)$$

is true. If it is true, then we can decompose after Grover’s algorithm on the subspace L

(25) $$\frac{1}{\sqrt{2^{m}}}\frac{1}{\sqrt{v}}\cdot \sum_{z\in B^{m}}\sum_{k=1}^{v}{\left(-1\right)}^{o(z,h_{k})}\cdot |z,h_{k}\rangle =$$

(26) $$\frac{1}{\sqrt{2^{m}}}\frac{1}{\sqrt{v}}\cdot \left(\sum_{z\in B^{m}}\sum_{h_{k}\neq l}{\left(-1\right)}^{o(z,h_{k})}\cdot |z,h_{k}{\rangle +\left(-1\right)}^{o(z,h_{k})}\cdot |\xi \rangle \right)=$$

(27) $$\frac{1}{\sqrt{2^{m}}}\frac{1}{\sqrt{v}}\cdot \sum_{z\in B^{m}}\sum_{h_{k}\neq l}|z,h_{k}\rangle -\frac{1}{\sqrt{2^{m}}}\frac{1}{\sqrt{v}}\cdot |\xi \rangle$$

After Grover’s search on the subspace U, if true,

(28) $$\frac{1}{\sqrt{v}}\cdot \left(|u\rangle ⊗\sum_{h_{k}\neq l}|h_{k}\rangle \right)+\frac{1}{\sqrt{v}}\cdot |\xi \rangle =$$

$$|u\rangle ⊗\left(\frac{1}{\sqrt{v}}\sum_{i=k}^{v}|h_{k}\rangle \right)$$

If not true, for v>2

$$\frac{1}{\sqrt{v-2}}\cdot |u\rangle ⊗\frac{1}{\sqrt{\left(v-2\right)\cdot 2^{m}}}\left(\sum_{z\in B^{m}}|z\rangle \right)⊗\left(\frac{1}{\sqrt{v}}\sum_{i=k}^{v}|h_{k}\rangle \right)$$

and for v=2

$$\frac{1}{\sqrt{2^{m}}}\left(\sum_{z\in B^{m}}|z\rangle \right)⊗\left(\frac{1}{\sqrt{v}}\sum_{i=k}^{v}|h_{k}\rangle \right).$$

In the event that |u〉 does not exist for any *z*

(29) $$\left(\frac{1}{\sqrt{2^{m}}}\frac{1}{\sqrt{v}}\cdot \sum_{z\in B^{m}}\sum_{k=1}^{v}|z,h_{k}\rangle \right)=\left(\frac{1}{\sqrt{2^{m}}}\sum_{z\in B^{m}}|z\rangle \right)⊗\left(\frac{1}{\sqrt{v}}\sum_{k=1}^{v}|h_{k}\rangle \right).$$

Since we do not know which $h_{k}=l$ we have to test all combinations, but we can reuse the set *ℵ* once computed.

$$\left(\frac{1}{\sqrt{2^{m}}}\frac{1}{\sqrt{v}}\cdot \sum_{z\in B^{m}}\sum_{h_{k}\neq h_{1}}{\left(-1\right)}^{o(z,h_{k})}\cdot |z,h_{k}\rangle +\frac{1}{\sqrt{2^{m}}}\frac{1}{\sqrt{v}}\cdot {\left(-1\right)}^{o(z,h_{k})}\cdot |\xi \rangle \right)$$

$$⊗\left(\frac{1}{\sqrt{2^{m}}}\frac{1}{\sqrt{v}}\cdot \sum_{z\in B^{m}}\sum_{h_{k}\neq h_{2}}{\left(-1\right)}^{o(z,h_{k})}\cdot |z,h_{k}\rangle +\frac{1}{\sqrt{2^{m}}}\frac{1}{\sqrt{v}}\cdot {\left(-1\right)}^{o(z,h_{k})}\cdot |\xi \rangle \right)$$

⋯⋯⋯⋯

(30) $$⊗\left(\frac{1}{\sqrt{2^{m}}}\frac{1}{\sqrt{v}}\cdot \sum_{z\in B^{m}}\sum_{h_{k}\neq h_{v}}{\left(-1\right)}^{o(z,h_{k})}\cdot |z,h_{k}\rangle +\frac{1}{\sqrt{2^{m}}}\frac{1}{\sqrt{v}}\cdot {\left(-1\right)}^{o(z,h_{k})}\cdot |\xi \rangle \right)=$$

$$\left(\frac{1}{\sqrt{2^{m}}}\frac{1}{\sqrt{v}}\cdot \sum_{z\in B^{m}}\sum_{h_{k}\neq h_{1}}|z,h_{k}\rangle -\frac{1}{\sqrt{2^{m}}}\frac{1}{\sqrt{v}}\cdot |\xi \rangle \right)$$

$$⊗\left(\frac{1}{\sqrt{2^{m}}}\frac{1}{\sqrt{v}}\cdot \sum_{z\in B^{m}}\sum_{h_{k}\neq h_{2}}|z,h_{k}\rangle -\frac{1}{\sqrt{2^{m}}}\frac{1}{\sqrt{v}}\cdot |\xi \rangle \right)$$

⋯⋯⋯⋯

(31) $$⊗\left(\frac{1}{\sqrt{2^{m}}}\frac{1}{\sqrt{v}}\cdot \sum_{z\in B^{m}}\sum_{h_{k}\neq h_{v}}|z,h_{k}\rangle -\frac{1}{\sqrt{2^{m}}}\frac{1}{\sqrt{v}}\cdot |\xi \rangle \right).$$

After Grover’s algorithm on the subspace U,

$$\left(\left(\frac{1}{\sqrt{v-2}}\cdot |u\rangle +\frac{1}{\sqrt{\left(v-2\right)\cdot 2^{m}}}\sum_{z\in B^{m}}|z\rangle \right)⊗\left(\frac{1}{\sqrt{v}}\sum_{i=k}^{v}|h_{k}\rangle \right)\right)$$

$$⊗\left(|u\rangle ⊗\left(\frac{1}{\sqrt{v}}\sum_{i=k}^{v}|h_{k}\rangle \right)\right)$$

⋯⋯⋯⋯

$$⊗\left(\left(\frac{1}{\sqrt{v-2}}\cdot |u\rangle +\frac{1}{\sqrt{\left(v-2\right)\cdot 2^{m}}}\sum_{z\in B^{m}}|z\rangle \right)⊗\left(\frac{1}{\sqrt{v}}\sum_{i=k}^{v}|h_{k}\rangle \right)\right)$$

and if *u* is not present we get a general superposition. We arrive at the decomposition

(32) $$H=U_{h_{k}\neq h_{1}}⊗U_{h_{k}\neq h_{2}}⊗\cdots ⊗U_{h_{k}\neq h_{v}}⊗L$$

We have determined the correct $h^{k}\left(y\right)$ by measuring |u〉 with a probability of approximately 1 in U. Subsequently, we can estimate the path descriptor $h_{k}=l$ using Grover’s algorithm on the subspace L. The probability measurement of $\frac{1}{v-2}$ indicates that $h^{k}\left(y\right)$ does not equal *l* and does not constitute a valid solution. However, for v>4, it necessitates a substantial number of measurements to ascertain that the probability deviates from 1.

### 5.1. Example with v=2

Our previous example with v=2

$$\left(\frac{1}{\sqrt{2^{3}}}\frac{1}{\sqrt{2}}\cdot \sum_{z\in B^{3}}{\left(-1\right)}^{o(z,h_{k})}\cdot |z,h_{1}\rangle +\frac{1}{\sqrt{2^{3}}}\frac{1}{\sqrt{2}}\cdot {\left(-1\right)}^{o(z,h_{k})}\cdot |\xi \rangle \right)$$

$$⊗\left(\frac{1}{\sqrt{2^{3}}}\frac{1}{\sqrt{2}}\cdot \sum_{z\in B^{3}}{\left(-1\right)}^{o(z,h_{k})}\cdot |z,h_{2}\rangle +\frac{1}{\sqrt{2^{3}}}\frac{1}{\sqrt{2}}\cdot {\left(-1\right)}^{o(z,h_{k})}\cdot |\xi \rangle \right)=$$

$$\left(\frac{1}{\sqrt{2^{3}}}\frac{1}{\sqrt{2}}\cdot \sum_{z\in B^{3}}|z,h_{1}\rangle -\frac{1}{\sqrt{2^{3}}}\frac{1}{\sqrt{2}}\cdot |\xi \rangle \right)$$

$$⊗\left(\frac{1}{\sqrt{2^{3}}}\frac{1}{\sqrt{2}}\cdot \sum_{z\in B^{3}}|z,h_{2}\rangle -\frac{1}{\sqrt{2^{3}}}\frac{1}{\sqrt{2}}\cdot |\xi \rangle \right).$$

After Grover’s algorithm on the subspace U we get the decomposition

(33) $$H=U_{h_{k}\neq h_{1}}⊗U_{h_{k}\neq h_{2}}⊗L=U_{h_{2}}⊗U_{h_{1}}⊗L,$$

with

$$\left(\frac{1}{2^{3}}\sum_{z\in B^{m}}|z\rangle \right)⊗\left(\frac{1}{2}\cdot \left(|\xi \rangle +\left|u\rangle \right|h_{2}\rangle \right)\right)⊗\left(\frac{1}{2}\cdot \left(|h_{1}\rangle +|h_{2}\rangle \right)\right)=$$

$$\left(\frac{1}{2^{3}}\sum_{z\in B^{m}}|z\rangle \right)⊗\left(\frac{1}{2}\cdot \left(\left|u\rangle \right|h_{1}\rangle +|u\rangle |h_{2}\rangle \right)\right)⊗\left(\frac{1}{2}\cdot \left(|h_{1}\rangle +|h_{2}\rangle \right)\right)=$$

$$\left(\frac{1}{2^{3}}\sum_{z\in B^{m}}|z\rangle \right)⊗|u\rangle ⊗\frac{1}{2}\cdot \left(|h_{1}\rangle +|h_{2}\rangle \right).$$

Because of the equality $|\xi \rangle =\left|u\rangle \right|h_{1}\rangle$ we know that $h^{1}\left(y\right)$ is correct and can estimate the path descriptor $h_{1}=l$ by Grover’s algorithm on the subspace L.

### 5.2. Costs

Within the subspace L, the cost associated with Grover’s algorithm is $\sqrt{\left(2^{m/2}/v\right)}$. For *v* combinations in the subspace U, the corresponding cost is $v\cdot \sqrt{2^{m/2}}$. Additionally, given $h_{k}$, we need to determine the path descriptor $h_{k}=l$ by Grover’s algorithm in the subspace L. The complete costs are

(34) $$\sqrt{\frac{2^{m/2}}{v}}+\sqrt{2^{m/2}}+v\cdot \sqrt{2^{m/2}}=\sqrt{2^{m/2}}\cdot \left(\frac{1}{\sqrt{v}}+1+v\right)$$

We can express the cost of iterative search in *O* notation, given that v≈m as before, with

$$O\left(v\cdot n^{1/4}\right)=O\left(\log n\cdot n^{1/4}\right).$$

For 1<v, the actual costs are less than the cost of the iterative algorithm with

(35) $$\sqrt{\frac{2^{m/2}}{v}}+\sqrt{2^{m/2}}+v\cdot \sqrt{2^{m/2}}<v\cdot \left(\sqrt{2^{m/2}}+\sqrt{2^{m/2}}+1\right)$$

(36) $$\sqrt{2^{m/2}}\cdot \left(\frac{1}{\sqrt{v}}+1\right)<v\cdot \sqrt{2^{m/2}}<v\cdot \left(\sqrt{2^{m/2}}+1\right)$$

(37) $$\frac{1}{\sqrt{v}}+1<v,$$

and they are cheaper in relation to times.

(38) $$times=\frac{v\cdot \left(2\cdot \sqrt{2^{m/2}}+1\right)}{\sqrt{2^{m/2}}\cdot \left(\frac{1}{\sqrt{v}}+1+v\right)},$$

For v>4, a substantial number of measurements is necessary. A practical approach would be to select $v^{*}=4$, resulting in approximately 1.45 faster costs

$$times=\frac{8\cdot \sqrt{2^{m/2}}+4}{\sqrt{2^{m/2}}\cdot 5.5}\approx 1.45$$

compared to the iterative approach with $v/4=v/v^{*}$ possible iterations.

#### Implementation

For the *ℵ* of *v* partial candidate solution functions that acts on *g* lower qubits on the subspace L

$$h^{\left(k\right)}\left(y\right)\;\;with\;k\in \left\{1,2,\cdots ,v\right\}$$

we introduce a $flag_{k}$ variable

(39) $$h^{\left(k\right)}\left(y\right)=\left\{\begin{array}{c} 1=flag_{k}\;\;\;if\;\;\;y=h_{k}\\ 0=flag_{k}\;\;\;else \end{array}\right.$$

and extend the upper oracle in relation to the $flag_{k}$ variable

(40) $${\tilde{u}}^{\left(k\right)}\left(z\right)=\left\{\begin{array}{c} 1\;\;\;if\;\;\;\left(z=u\right)\wedge \left(flag_{k}=1\right)\\ 0\;\;\;else \end{array}\right.$$

For example, for the case $l=h_{s}$, ${\tilde{u}}^{\left(s\right)}\left(z\right)=o_{\xi }\left(x\right)$. To determine a possible solution we have to measure *v* subspaces U.

**Figure 10:**
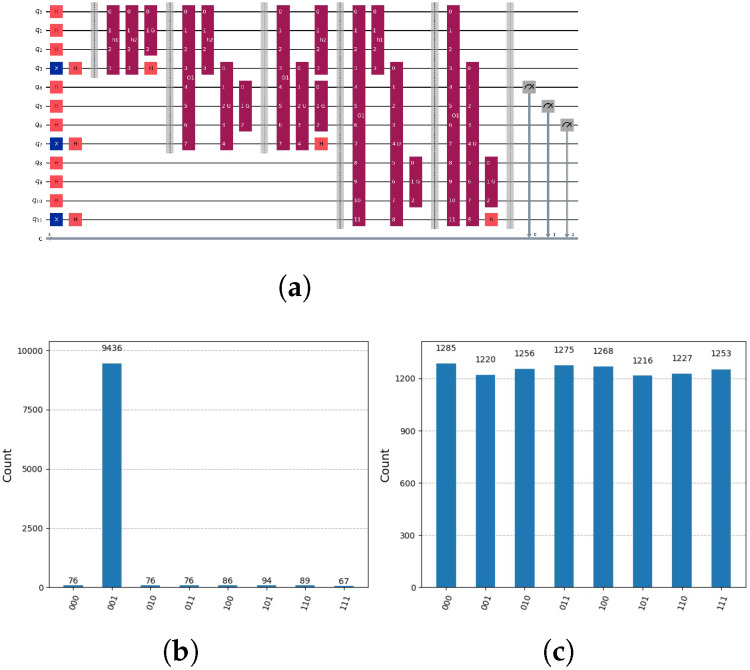
(**a**) Circuit employing Grover’s amplification to represent oracles $h^{\left(1\right)}\left(z\right)=\neg x_{3}\wedge x_{2}\wedge x_{1}$, $h^{\left(2\right)}\left(z\right)=x_{3}\wedge \neg x_{2}\wedge x_{1}$ representing the decomposition $U_{h_{2}}⊗U_{h_{1}}⊗L$. The subspace L is represented by the qubits 0–2, subspace $U_{h_{1}}$ is represented by the qubits 4–6 and subspace $U_{h_{2}}$ is represented by the qubits 8–10. Notation: *H* indicates Hadamard gate, *X* indicates NOT Gate, *h1* and *h2* partial candidate solution circuits that marks the solution in the auxiliary qubits. *O* represents the global oracle circuits and *G* Grover’s algorithm circuits. Composed quantum circuits are indicated dark red, quantum gates with colours orange and blue. Notation: *H* indicates Hadamard gate, *X* indicates NOT Gate, *h1* and *h2* partial candidate solution circuits that marks the solution in the auxiliary qubits. *O* represents the global oracle circuits, *U* upper part of the oracle function and *G* Grover’s algorithm circuits. Composed quantum circuits are indicated dark red, quantum gates with colours orange and blue. (**b**) We measure the subspace $U_{h_{1}}$ representing the solution. (**c**) We measure the subspace $U_{h_{2}}$ representing a non-solution.

For example, in the preceding SAT example with v=2 and g=3 we introduce the $flag_{k}$ variable

$$h^{\left(1\right)}\left(y\right)=\neg x_{3}\wedge x_{2}\wedge x_{1},\;\;h^{\left(2\right)}\left(z\right)=x_{3}\wedge \neg x_{2}\wedge x_{1}$$

$$u{\left(z\right)}^{\left(k\right)}=\left(\neg x_{6}\wedge \neg x_{5}\wedge x_{4}\right)\wedge flag_{k}$$

$$o\left(x\right)=\neg x_{6}\wedge \neg x_{5}\wedge x_{4}\wedge \neg x_{3}\wedge x_{2}\wedge x_{1}$$

where $o_{\xi }\left(x\right)$ corresponds to $u{\left(y\right)}^{1}$. The decomposition

$$H=U_{h_{2}}⊗U_{h_{1}}⊗L,$$

is represented by the quantum circuit in Figure 10a.

## 6. Permutation in the Subspace L

**Figure 11:**
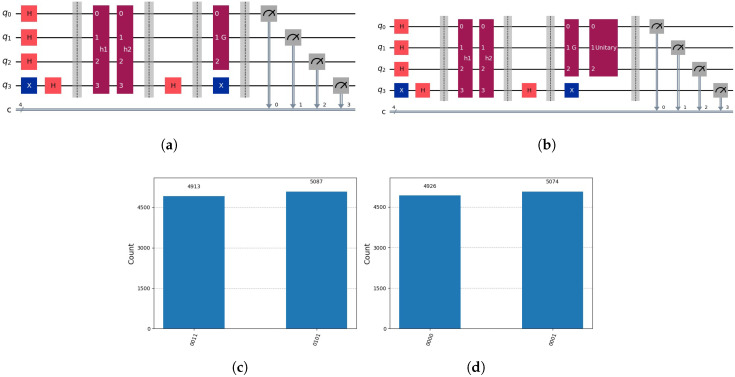
(**a**) After applying Grover’s algorithm, with two solutions indicated by $h^{1}\left(y\right)$ and $h^{2}\left(y\right)$. (**b**) Mapping by permutation operator *P* indicated by the gate “Unitary” of the register $|y\rangle =\frac{1}{\sqrt{2}}\cdot (|011\rangle +\left|101\rangle \right)$ into $|x\rangle =|00\rangle ⊗\frac{1}{\sqrt{2}}\cdot (|0\rangle +\left|1\rangle \right)$. Notation: *H* indicates Hadamard gate, *X* indicates NOT Gate, *h1* and *h2* partial candidate solution circuits that marks the solution in the auxiliary qubits. *G* indicates Grover’s algorithm circuits and *Unitary* the permutation operator *P* circuit. Composed quantum circuits are indicated dark red, quantum gates with colours orange and blue. (**c**) Measuring the circuit (**a**). (**d**) Measuring the circuit (**b**) representing $P\cdot \left(\frac{1}{\sqrt{2}}\cdot (|011\rangle +\left|101\rangle \right)\right)=|00\rangle ⊗\frac{1}{\sqrt{2}}\cdot (|0\rangle +\left|1\rangle \right)$.

We can map the set of solutions determined by the function of the set *ℵ* into in the subspace L into another ordering that uses only the corresponding subset that could lead to a decomposition. For example, the superposition

$$\frac{1}{\sqrt{2}}\cdot (|011\rangle +\left|101\rangle \right)$$

is mapped by a permutation matrix *P*

$$P\cdot \left(\frac{1}{\sqrt{2}}\cdot (|011\rangle +\left|101\rangle \right)\right)=\frac{1}{\sqrt{2}}\cdot (|000\rangle +\left|001\rangle \right)=$$

(41) $$|00\rangle ⊗\frac{1}{\sqrt{2}}\cdot (|0\rangle +\left|1\rangle \right)$$

into the decomposition $|00\rangle ⊗\frac{1}{\sqrt{2}}\cdot (|0\rangle +\left|1\rangle \right)$, see Figure 11.

We can represent the permutation matrix *P* as

$$\left(\begin{array}{c} 1\\ 1\\ 0\\ 0\\ 0\\ 0\\ 0\\ 0 \end{array}\right)=\left(\begin{array}{cccccccc} 0 & 0 & 0 & 1 & 0 & 0 & 0 & 0\\ 0 & 0 & 0 & 0 & 0 & 1 & 0 & 0\\ 0 & 0 & 1 & 0 & 0 & 0 & 0 & 0\\ 1 & 0 & 0 & 0 & 0 & 0 & 0 & 0\\ 0 & 0 & 0 & 0 & 1 & 0 & 0 & 0\\ 0 & 1 & 0 & 0 & 0 & 0 & 0 & 0\\ 0 & 0 & 0 & 0 & 0 & 0 & 1 & 0\\ 0 & 0 & 0 & 0 & 0 & 0 & 0 & 1 \end{array}\right)\cdot \left(\begin{array}{c} 0\\ 0\\ 0\\ 1\\ 0\\ 1\\ 0\\ 0 \end{array}\right).$$

The operation corresponds to the mapping by permutation operator *P* of a register $|y_{k}\rangle$ of *m* qubits into a register $|x_{k}\rangle$ of *m* qubits, of which the first *g* qubits are ${|0\rangle }^{⊗g}$, with

(42) $$\frac{1}{\sqrt{v}}\sum_{k=1}^{v}P\cdot |y_{k}{\rangle =|0\rangle }^{⊗g}⊗\left(\frac{1}{\sqrt{v}}\sum_{k=1}^{v}|x_{k}\rangle \right).$$

Since Qiskit uses little-endian notation we indicate the inverse order by a permutation matrix $P^{*}$

$$P^{*}\cdot \left(\frac{1}{\sqrt{2}}\cdot (|011\rangle +\left|101\rangle \right)\right)=\frac{1}{\sqrt{2}}\cdot (|000\rangle +\left|100\rangle \right)=$$

(43) $$\frac{1}{\sqrt{2}}\cdot (|0\rangle +\left|1\rangle \right)⊗|00\rangle$$

into the decomposition $\frac{1}{\sqrt{2}}\cdot (|0\rangle +\left|1\rangle \right)⊗|00\rangle$. We can represent the permutation matrix P∗, and due to little-endian notation we get

$$\left(\begin{array}{c} 1\\ 0\\ 0\\ 0\\ 1\\ 0\\ 0\\ 0 \end{array}\right)=\left(\begin{array}{cccccccc} 0 & 0 & 0 & 1 & 0 & 0 & 0 & 0\\ 0 & 1 & 0 & 0 & 0 & 0 & 0 & 0\\ 0 & 0 & 1 & 0 & 0 & 0 & 0 & 0\\ 1 & 0 & 0 & 0 & 0 & 0 & 0 & 0\\ 0 & 0 & 0 & 0 & 0 & 1 & 0 & 0\\ 0 & 0 & 0 & 0 & 1 & 0 & 0 & 0\\ 0 & 0 & 0 & 0 & 0 & 0 & 1 & 0\\ 0 & 0 & 0 & 0 & 0 & 0 & 0 & 1 \end{array}\right)\cdot \left(\begin{array}{c} 0\\ 0\\ 0\\ 1\\ 0\\ 1\\ 0\\ 0 \end{array}\right).$$

The operation corresponds to the mapping by permutation operator $P^{*}$ of a register $|y_{k}\rangle$ of *m* qubits into a register $|x_{k}\rangle$ of *m* qubits, of which the last *g* qubits are ${|0\rangle }^{⊗g}$, with

(44) $$\frac{1}{\sqrt{v}}\sum_{k=1}^{v}P^{*}\cdot |y_{k}\rangle =\left(\frac{1}{\sqrt{v}}\sum_{k=1}^{v}|x_{k}\rangle \right)⊗{|0\rangle }^{⊗g}.$$

**Figure 12:**
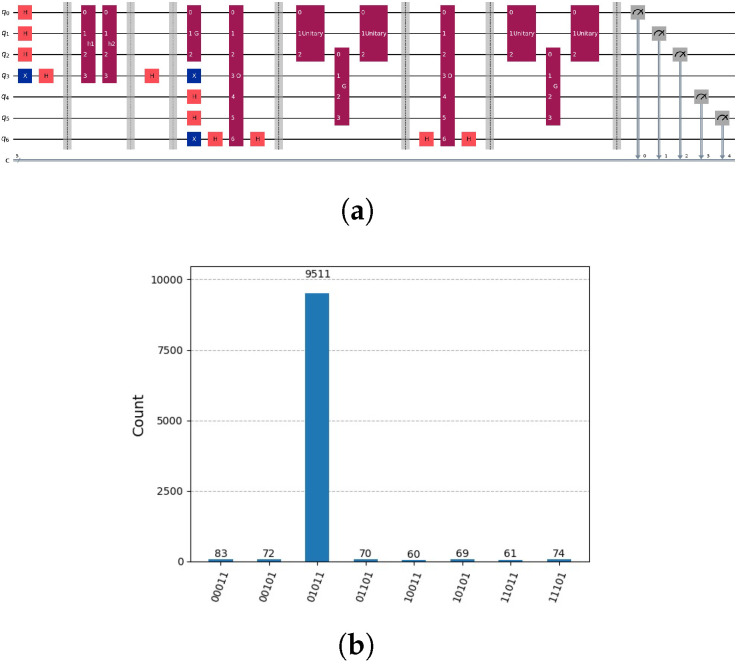
(**a**) The corresponding circuit; the permutation operator P∗ is indicated by the gate “Unitary”. After applying Grover’s algorithm with two solutions indicated by $h^{1}\left(y\right)$ and $h^{2}\left(y\right)$, using the oracle on the original space $\frac{1}{\sqrt{2}}\cdot (-|011\rangle +\left|101\rangle \right)$ to mark the solution and mapping $P^{*}\cdot \left(\frac{1}{\sqrt{2}}\cdot (-|011\rangle +\left|101\rangle \right)\right)=\frac{1}{\sqrt{2}}\cdot (-|0\rangle +\left|1\rangle \right)⊗|00\rangle$. Qubit 2 indicates the marked solution describing the subspace L by permuted values $h_{1}$ and $h_{2}$. Qubits 4 and 5 correspond to the subspace U. Grover’s algorithm is applied on the qubits 2, 4 and 5. We the transpose $P^{*}$ to determine the solution by the oracle $o_{\xi }\left(x\right)$ in the original space. This is quite elegant, but until now, there appears to be no straightforward method to ascertain the mapping represented by $P^{*}$. Notation: *H* indicates Hadamard gate, *X* indicates NOT Gate, *h1* and *h2* partial candidate solution circuits that marks the solution in the auxiliary qubits. *O* represents the global oracle circuits, *G* indicates Grover’s algorithm circuits and *Unitary* the permutation operator *P* circuit. Composed quantum circuits are indicated dark red, quantum gates with colours orange and blue. (**b**) Determining the correct solution in the original space.

We can apply such a permutation operator $P^{*}$ for a nested Grover’s algorithm for tree search, as indicated in Figure 12. Grover’s algorithm is applied on the qubits 2, 4 and 5.

### 6.1. Costs

Ignoring the costs of the permutation operator *P* with the preparation costs *v* by basis encoding in subspace L, as described by [20,21,22], the costs corresponding to the Grover’s algorithm applied to $m/2+\log_{2}v$ qubits are

(45) $$v+\sqrt{2^{m/2+\log_{2}v}}=v+\sqrt{2^{m/2}}\cdot \sqrt{v}.$$

With the preparation costs $\sqrt{\frac{2^{m/2}}{v}}$ using Grover’s algorithm in subspace L the costs are

(46) $$\sqrt{\frac{2^{m/2}}{v}}+\sqrt{2^{m/2+\log_{2}v}}=\sqrt{2^{m/2}}\cdot \left(\frac{1}{\sqrt{v}}+\sqrt{v}\right)$$

in *O* notation, given v≈m, with

$$O\left(v^{1/2}\cdot n^{1/4}\right)=O\left({\left(\log n\right)}^{1/2}\cdot n^{1/4}\right).$$

### 6.2. Permutation Operator P

**Figure 13:**
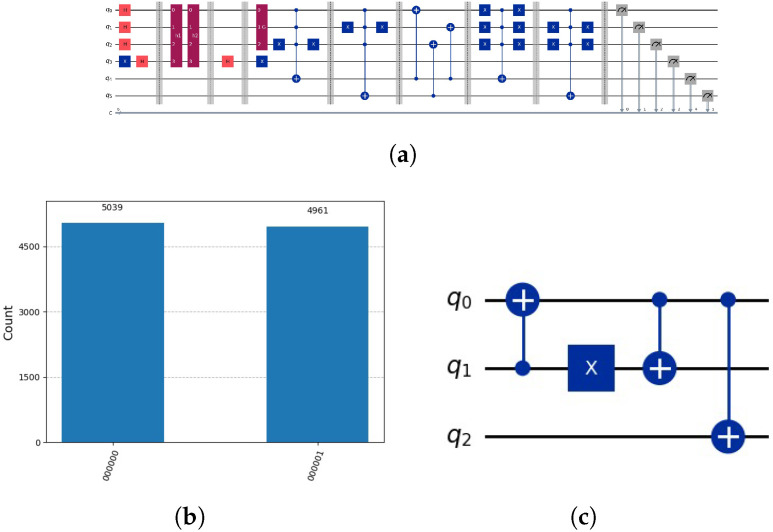
(**a**) Representation of the permutation operator $P\cdot \left(\frac{1}{\sqrt{2}}\cdot (|011\rangle +\left|101\rangle \right)\right)=|00\rangle ⊗\frac{1}{\sqrt{2}}\cdot (|0\rangle +\left|1\rangle \right)$ by controlled not gates. First we indicate the two paths $h_{k}$ in superposition by two flag qubits 4 and 5. We used the two flag qubits and the knowledge of the two paths $h_{1},h_{2}$ to perform the permutation mapping. Knowing the permutation mapping, we disentangle the two flag qubits 4 and 5 by mapping them to zero. Notation: *H* indicates Hadamard gate, *X* indicates NOT Gate and is used in as well in controlled not gates, *h1* and *h2* partial candidate solution circuits that marks the solution in the auxiliary qubits. *G* indicates Grover’s algorithm circuit. (**b**) The correct mapping $|00\rangle ⊗\frac{1}{\sqrt{2}}\cdot (|0\rangle +\left|1\rangle \right)$ without entanglement of the additional qubits 3, 4 and 5 that are mapped to 0. (**c**) A compressed representation of the permutation operator *P*.

There appears to be no straightforward method to ascertain the mapping represented by *P*. The permutation operator *P* is defined by the *v* paths $h_{k}$ to partial could-be solutions. Due to entanglement we cannot determine the operator *P* using $h^{k}\left(z\right)$ and the resulting $h_{k}$ paths. Due to entanglement we cannot determine the permutation operator *P* without knowing the $h_{k}$ paths that indicate which nodes indicate partial could-be solutions. Knowing the $h_{k}$ paths we can determine the permutation operator *P* by controlled not gates, see Figure 13a. Using the regularities, permutation operator *P* can be considerably compressed, see Figure 13c, leading to reduced costs.

Knowing $h_{k}$ paths defines distinct nodes of the same depth simultaneously. For instance, in the quantum search tree depicted in Figure 6, the two extended oracles would be $o_{1}\left(011,u\right)$ and $o_{2}\left(000,u\right)$, with g=3, and we would seek the solution *u*. This approach is appropriate for a tree search where we presume that certain nodes at a depth *g* signify potential solutions. Alternatively, we could implement the two extended oracles directly through a quantum circuit using concatenation.

## 7. Discussion

### 7.1. Partial Candidate Solution h(y)

Within the context of quantum tree search, instead of employing a heuristic function μ(y), we will utilize the function h(y), which signifies a potential solution, and *y* represents a path from the root to the corresponding node. In contrast to the heuristic function μ(y), which can mark multiple nodes, h(y) should exclusively mark a single potential solution for the node. A set of partial candidate solutions can be identified by determining their similarity or distance to the goal state. However, how can we pinpoint a single potential solution, a single partial candidate solution? One approach to achieve this is to introduce an additional constraint that narrows the set to a single function, denoted by $h_{k}\left(y\right)$. Such a constraint could, for instance, specify the possible position within a subtree, thereby reducing the set to a single function. It is important to note that this approach is highly probabilistic and may occasionally yield non-existent partial candidate solutions or values exceeding one. Additionally, we have to take care that *v* is a constraint, as indicated by the Equation (23). In addition, further research should investigate the partial candidate solution $h_{k}\left(y\right)$ function on examples, as indicated in the paper [5].

### 7.2. Generalized Quantum Tree Search

We must have prior knowledge of the depth *m* off the search tree. This constraint can be circumvented through iterative deepening. In an iterative deepening search, we progressively increase the search limit from one to two to three to four and continue until a goal is discovered. For each limit, a search is conducted from the root to the maximum depth of the search tree. Should the search prove unsuccessful, a new search is initiated with a deeper limit. During the iterative deepening search, states are generated multiple times [23,24]. The time complexity of an iterative deepening search is comparable to that of a search to the maximum depth [23]. A quantum iterative deepening search is equivalent to an iterative deepening search [6]. For each limit max, a quantum tree search is performed from the root, where max represents the maximum depth of the search tree. The potential solutions are determined through measurement.

For a non-constant branching factor, the maximal branching factor $B_{max}$ has to be used for the quantum tree search [6]. For the maximum value of $B_{max}$, the quantum algorithm using qubit representation outperforms the classical tree search described by the effective branching factor *b* in the specified case

(47) $$b>b_{q}=\sqrt{B_{max}}.$$

For a large number of instances with varying initial and goal states, the effective branching factor converges to the average branching factor for an uninformed tree search, as shown in [5].

Consider each branching factor as a potential local path. For a non-constant branching factor, the quantum tree search utilizes the maximal branching factor, denoted by $B_{max}$. If, in a node ν, $B_{\nu }<B_{max}$, only $B_{\nu }$ are required, and $B_{max}-B_{\nu }$ local paths are not utilized. To address this limitation, we augment the path descriptor by repeating the paths. This approach ultimately leads to *k* solutions that converge to the effective branching factor, as demonstrated in [5].

## 8. Conclusions

A primary outcome of the investigation is that conventional heuristic functions are unsuitable for quantum tree search due to the inability to disentangle the subspaces utilized by Grover’s algorithm. Furthermore, the theoretical concepts of the potential permutations within the subspace L suggest that due to entanglement, we cannot determine the permutation operator *P* without knowing the paths that represent the values of the heuristic functions.

Consequently, only partial candidate solutions possess the capability to disentangle these subspaces. We assume the existence of a set of partial candidate solutions, denoted by *ℵ*, that can be determined, for instance, by their similarity to the goal state. We impose the constraint that within this set, only one maximal partial candidate solution, denoted by $h_{k}\left(y\right)$, identifies a potential solution. Each partial candidate solution of the set *ℵ* can be constrained to be valid only in a specific subtree. It is important to note that a partial candidate solution is not a position in the search tree itself, as its values are unknown and are determined by Grover’s algorithm and may occasionally yield non-existent partial candidate solutions or values exceeding one candidate solution.

With the existence of the partial candidate solution h(y), the iterative approach represents a considerable speedup with the costs

(48) $$v\cdot \left(\sqrt{2^{m/2}}+\sqrt{2^{m/2}}+1\right)=v\cdot \left(2\cdot 2^{m/4}+1\right)$$

We can express the cost of iterative search it in *O* notation, given v≈m, since $n=2^{m}$ as

$$O\left(v\cdot n^{1/4}\right)=O\left(\log n\cdot n^{1/4}\right).$$

Combinatory approach with the possible existence of the upper oracle u(z) allows us to disentangle L and U using

$$H=U_{h_{k}\neq h_{1}}⊗U_{h_{k}\neq h_{2}}⊗\cdots ⊗U_{h_{k}\neq h_{v}}⊗L$$

which speeds up the costs minimally.

The work suggests a novel direction based on a partial candidate solution rather than heuristic functions that are unsuitable for quantum tree search. All Qiskit (v2.2) examples are presented in a Jupyter Notebooks that can be freely downloaded at https://github.com/andrzejwichert/Nested-Quantum-Tree-Search (accessed on 16 December 2025).

## Figures and Tables

**Figure 4:**
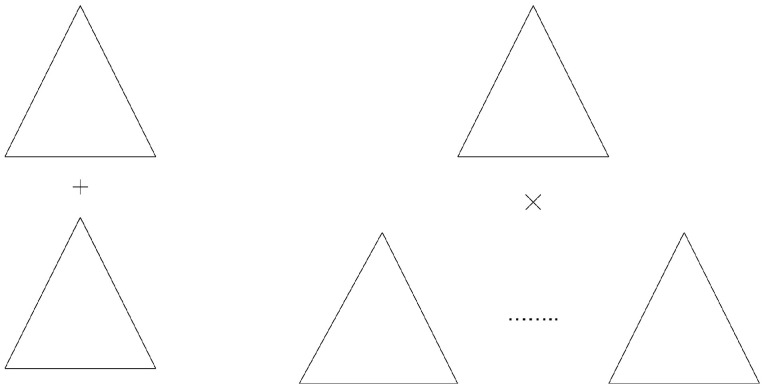
The concept of cost savings is predicated on the inequality that decomposes the Hilbert space H of dimension $n=2^{m}$ into subspaces H=U⊗L of dimensions $2^{g}$ and $2^{m-g}$, respectively. On the left side, the sum of the costs of Grover’s algorithm, with leaves for addition resulting in $\sqrt{2^{g}}+\sqrt{2^{m-g}}$, is represented. On the right side, the original costs of Grover’s algorithm are $\sqrt{2^{m}}=2^{g}\cdot 2^{m-g}$, corresponding to the multiplication operation.

## Data Availability

The data presented in this study are openly available in [Nested-Quantum-Tree-Search] at https://github.com/andrzejwichert/Nested-Quantum-Tree-Search (accessed on 16 December 2025).
